# Effects of Calcium Lactate-Enriched Pumpkin on Calcium Status in Ovariectomized Rats

**DOI:** 10.3390/foods11142084

**Published:** 2022-07-13

**Authors:** Natalia Wawrzyniak, Anna Gramza-Michałowska, Ewa Pruszyńska-Oszmałek, Maciej Sassek, Joanna Suliburska

**Affiliations:** 1 Department of Human Nutrition and Dietetics, Faculty of Food and Nutrition Science, University of Life Sciences, 60-624 Poznan, Poland; natalia.wawrzyniak@up.poznan.pl; 2 Department of Gastronomy Sciences and Functional Foods, Faculty of Food and Nutrition Science, University of Life Sciences, 60-624 Poznan, Poland; anna.gramza@up.poznan.pl; 3 Department of Animal Physiology and Biochemistry, Faculty of Veterinary Medicine and Animal Science, University of Life Sciences, 60-637 Poznan, Poland; ewa.pruszynska@up.poznan.pl (E.P.-O.); maciej.sassek@up.poznan.pl (M.S.)

**Keywords:** calcium, enriched pumpkin, postmenopausal osteoporosis, kidney accumulation

## Abstract

This study aimed to evaluate the effects of enriched pumpkin on calcium status in ovariectomized rats. The study was conducted in sixty female Wistar rats, which were divided into six groups: a group fed a standard diet (C) and five ovariectomized groups fed a standard diet (OVX_C) or a diet with calcium lactate (CaL), with calcium lactate-enriched pumpkin (P_CaL), with calcium lactate and alendronate (CaL_B), or with calcium lactate-enriched pumpkin with alendronate (P_CaL_B). After 12 weeks of the intervention, the rats were sacrificed, and their blood and tissues were collected. The calcium concentrations in serum and in tissues were measured using flame atomic absorption spectrometry (AAS). Serum concentrations of procollagen type-1 amino-terminal propeptide (PINP), parathyroid hormone PTH, estrogen (ES), and osteocalcin (OC) were determined with enzyme-linked immunosorbent assay (ELISA). It was found that enriched pumpkin increased the calcium level in the kidneys (194.13 ± 41.01 mg) compared to the C (87.88 ± 12.42 mg) and OVX_C (79.29 ± 7.66 mg) groups. The addition of alendronate increased the calcium level in the femurs (267.63 ± 23.63 mg) and more than six times in the kidneys (541.33 ± 62.91 mg) compared to the OVX_C group (234.53 ± 21.67 mg and 87.88 ± 12.42 mg, respectively). We found that the CaL, P_CaL, and CaL_B groups had significantly lower PINP serum concentrations (4.45 ± 0.82 ng/mL, 4.14 ± 0.69 ng/mL, and 3.77 ± 0.33 ng/mL) and higher PTH serum levels (3.39 ± 0.54 ng/dL, 3.38 ± 0.57 ng/dL, and 3.47 ± 0.28 ng/dL) than the OVX_C group (4.69 ± 0.82 ng/mL and 2.59 ± 0.45 ng/dL, respectively). In conclusion, pumpkin enriched with calcium lactate affects calcium status and normalizes PINP and PTH serum levels in ovariectomized rats. Diet with enriched pumpkin and alendronate increase calcium concentration in the femur. Enriched pumpkin causes calcium to accumulate in the kidneys of ovariectomized rats; alendronate significantly exacerbates this effect.

## 1. Introduction

Epidemiological studies have shown an inadequate dietary calcium intake, which may increase the risk of developing osteoporosis, especially in postmenopausal women. Natural menopause and ovariectomy lead to decreases in estrogen level in the body, as well as malabsorption of calcium, and this contributes to bone loss [1,2,3]. The adequate supply and bioavailability of calcium are important for bone health, as are endogenous factors regulating the calcium metabolism. Among the hormones related to calcium metabolism and bone turnover are osteocalcin (OC), procollagen type-1 amino-terminal propeptide (PINP), and parathyroid hormone (PTH) [4,5,6]. PINP and OC are involved in bone and dentin mineralization, which means that they are byproducts of the bone formation process. The release of osteocalcin from osteoblasts is stimulated by PTH, which is affected by serum calcium concentration, while PINP has become a reference marker in the diagnosis of osteoporosis, reflecting the process of collagen formation [5,6]. PTH is a hormone involved in calcium metabolism and bone remodeling. In the presence of sufficient serum calcium, extracellular calcium binds to receptors on parathyroid cells, reducing PTH levels. These mechanisms work in a feedback loop, meaning that PTH levels increase in hypocalcemia. The function of PTH is to increase serum calcium concentration though reabsorption from the kidneys, bone resorption, and intestinal absorption (by stimulating the production of active vitamin D in the kidneys) [7].

Bone loss characteristic of menopausal women is treated pharmacologically, mainly with bisphosphonates (e.g., alendronate) [8]. Alongside pharmacological therapy, a diet containing adequate amounts of good bioavailable calcium is part of osteoporosis treatment and prevention [9]. Dietary recommendations range from 1000 to 1500 mg of calcium/day, depending on age [10]. The most bioavailable calcium comes from dairy products; however, some people may suffer from lactose intolerance and cannot eat such kinds of products. Some plant products are also a good source of calcium, but it is less bioavailable [11]. Decreased intestinal absorption leads to the insufficient transport of calcium ions into the blood; thus, bioavailability is a critical factor that determines the effectiveness of calcium for bone development and health [12]. On the other hand, an excessive supply of calcium (mainly caused by taking calcium supplements) can result in many diseases, including renal failure, adrenal insufficiency, or hyperparathyroidism [13]. In order for the calcium contained in nutrients to be effectively absorbed in the intestines, factors that reduce its bioavailability should be avoided; these include iron, oxalic acid, and phytic acid. Conversely, factors that improve its bioavailability should be increased; these include vitamin D3, fructooligosaccharides, and inulin [14].

It is known that the bioavailability of calcium depends on its chemical form. Calcium lactate is an organic salt of calcium with a relatively high bioavailability and good solubility [15]. Calcium lactate is a component often used in supplements recommended for menopausal women [16,17]. An effective and easy way to increase the amount of calcium in the diet is through functional foods that contain much more calcium than natural foods [18]. One such innovative food is pumpkin enriched with calcium through osmotic dehydration using inulin, an osmotically active substance that increases calcium absorption [19]. Pumpkin is easy to use in an osmotic dehydration process leading to the enrichment of its tissues with calcium salts [19]. Moreover, pumpkin contains compounds that increase bone mineral density. Lutein is a carotenoid found abundantly in pumpkin which has been found to increase the mineral mass of bones, while suppressing their resorption by inhibiting the formation of osteoclasts [20,21]. Lutein also reduces the oxidative stress that accompanies postmenopausal osteoporosis [22]. Another carotenoid found in pumpkin is β-cryptoxanthin, which inhibits bone resorption and has an osteogenic effect through its effects on the expression of genes of proteins that are involved in bone formation [23]. Pumpkin might be widely used due to its low caloric content (average 26 kcal/100 g). Hypoglycemic and cardioprotective properties of pumpkin have been found; therefore, its consumption is recommended for people with hypertension, obesity, and diabetes. Moreover, due to the possibility of preparing dishes with a soft consistency, pumpkin is suitable both for infants, the elderly, and patients with gastrointestinal diseases [24].

Pumpkin has been enriched with calcium compounds using inulin, an osmotically active substance that increases calcium absorption, by changing the composition of the intestinal microbiota (increasing *Bifidobacterium*) [25], increasing the area of absorption in the cecum [26,27], and altering pH through the increased production of short-chain fatty acids [28]. It seems that consuming calcium-enriched pumpkin with inulin may be beneficial for people at high risk of osteoporosis development. Therefore, because pumpkin is a low-calorie food and a source of compounds with a high biological activity with a beneficial effect on the bone mineral status, and because it is easy to use in an osmotic dehydration process, we believe that a combination of pumpkin with calcium lactate may be a good source of high bioavailable calcium in prevention and treatment of postmenopausal osteoporosis. This study, thus, aimed to determine the effects of calcium-enriched pumpkin on calcium status in an animal model of postmenopausal osteoporosis.

## 2. Materials and Methods

### 2.1. Materials and Reagents

Calcium lactate and inulin were purchased (Agnex, Białystok, Poland), as were pumpkins sourced from domestic organic farming. Experimental research and field studies on plants, including the collection of plant material, complied with relevant national, institutional, and international guidelines and legislation. Permission to collect the plant material (pumpkin) was obtained from the landowner. The ingredients of the animal feed—vitamins, minerals, l-cysteine, and choline—were purchased from Sigma-Aldrich (Darmstadt, Germany), while casein, corn starch, dextrin, cellulose, sucrose, and rapeseed oil were purchased from Hortimex (Konin, Poland). ELISA (enzyme-linked immunosorbent assay) kits were purchased from Mediagnost (Reutlingen, Germany).

### 2.2. Osmotic Dehydration

The pumpkin tissue was enriched with calcium lactate using a process of osmotic dehydration with inulin, an osmotically active substance. During osmotic dehydration, the exchange of components between the solution and the pumpkin takes place; the water is removed from the pumpkin and the compounds dissolved in the solution (inulin and calcium lactate) penetrate the pumpkin tissue. Thus, the purpose of osmotic dehydration of pumpkin was to saturate its tissues with calcium and inulin so that it became a source of these ingredients.

At first, the pumpkin was washed and cleaned, and the inner part attached to the pips was removed. The skin was then removed, and the pumpkin flesh was cut into cuboids (1 cm), which then underwent osmotic dehydration. Before the next stage, the pumpkin was frozen to −18 °C and stored for 24 h for further analysis. A 50% solution of inulin was prepared in small jars, containing 125 mL of distilled water and 125 g of inulin; calcium lactate was added to give a concentration of 5%. The frozen pumpkin cubes were added to this hypertonic solution at a ratio of 1:5 (50 g of pumpkin and 250 g of solution); the jars were tightly closed and shaken in a water bath heated to 50 °C for 2 h. After the osmotic dehydration, the supernatant solution was removed, and the pumpkin was filtered. The entire procedure was performed in three replications. Before the freeze-drying process, the pumpkin was frozen to between −18 °C and −28 °C for 24 h. The drained pumpkin was dried in a freeze dryer until the water content reached 3.5–5%. There was 2797.00 ± 11.90 mg of calcium in 100 g of the resulting lyophilizate. The calcium content in the lyophilizate was determined using flame atomic absorption spectrometry (AAS-3, Carl Zeiss, Jena, Germany) after mineralization and dilution with deionized water and LaCl3 (0.5%).

### 2.3. Animals

Twelve week old female Wistar rats were purchased from the Wielkopolska Center for Advanced Technologies, Adam Mickiewicz University (Poznań, Poland). The animals were housed under standard conditions: single-caged, with a 12 h light–dark cycle, acclimated for 1 week. This animal experiment was carried out in accordance with the guidelines for the care and use of laboratory animals.

### 2.4. Experimental Protocols

The study was conducted on sixty rats. All rats were fed the AIN-93 M diet [29]. The animals were randomized into six groups, with 10 animals in each group. At the start of the experiment, the total body weight of the rats did not differ between groups. Each rat was housed in a separate cage that was set up so that the rats could see each other, reducing the stress associated with the study. At the start of the experiment, 50 rats were ovariectomized (OVX) to establish a rat model of postmenopausal osteoporosis.

**Figure 1:**
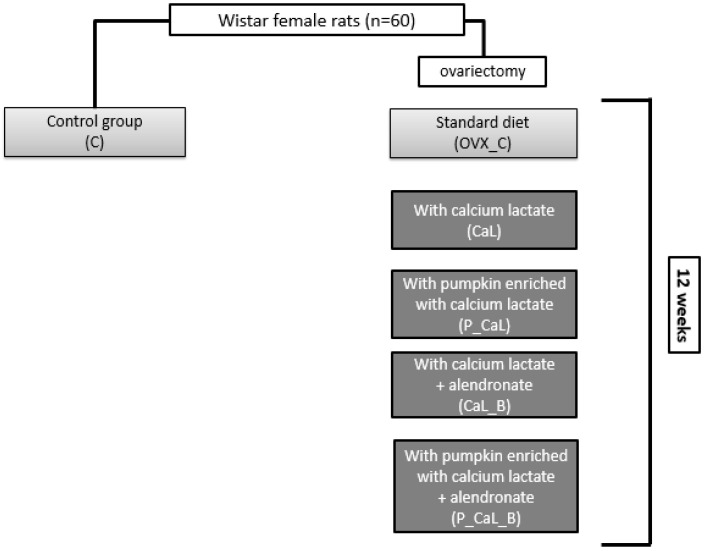
The scheme of the study. Groups shown in light-gray boxes were given the standard diet; the basic diet of the groups shown in the dark-gray boxes was a diet without calcium.

After 7 days of convalescence, the nutritional intervention was started for 12 weeks. The control group (C) and one of the ovariectomized groups (OVX_C) received standard diet (no modification), the CaL group was fed standard diet with calcium carbonate replaced by calcium lactate, the P_CaL group was fed pumpkin enriched with calcium lactate, the CaL_B group was fed alendronate (a drug from the group of bisphosphonates) and calcium lactate, and the P_CaL_B group was fed alendronate and pumpkin enriched with calcium lactate. The standard diet included (per kg of diet) 465.7 g of cornstarch, 155 g of dextrin, 140 g of casein, 100 g of sucrose, 50 g of fiber, 40 g of sunflower oil, 35 g of mineral mix, 10 g of vitamin mix, 2.5 g of choline bitartrate, and 1.8 g of l-cysteine. The amounts of calcium lactate (27.22 g) and enriched pumpkin (180 g) in 1 kg of the diet were such that the calcium content was the same as in the standard diet. In the diets with bisphosphonate, the potassium alendronate amount was adjusted weekly as needed to maintain a dose of 3 mg per kilogram of body weight. The scheme of the experiment is shown in Figure 1.

The animals were allowed to eat and drink deionized water ad libitum throughout the experiment. The rats in each group were weighed weekly, and food consumption was recorded daily. After completion of the experiment, body composition analysis of all animals was performed on a Bruker LF90II Body Composition Analyzer. The rats in each group were then decapitated, and blood and tissue samples were collected. Blood was centrifuged at 1200× *g* for 10 min at 4 °C. Livers, spleens, kidneys, pancreases, femurs, thigh muscles, and hair were removed, washed in saline, weighted, and kept at −80 °C for analysis. Hair was collected from the same anatomic area of each rat (the interscapular region).

### 2.5. Diet Analysis

The chemical composition of the diets included proteins, lipids, ash, carbohydrates, and fiber. The protein content was determined using the Kjeldahl method (AOAC, 1995; Foss Tecator, Höganäs, Sweden). The lipid content was determined using the Soxhlet method (PN-EN ISO 3947:2001; Soxtec System, Foss Tecator, Höganäs, Sweden). Ash content was determined after completely burning the sample in an oven (AOAC, 2000, Termo Fisher Scientific, Waltham MA, USA). Carbohydrate content was determined as 100% minus the percentage contributions of protein, fat, water, and ash. The fiber fractions were measured as total (TDF), insoluble (IDF), and soluble (SDF) dietary fiber content, following the enzymatic–gravimetric Asp method [30]. Other fiber fractions were evaluated using the Van Soest Assay [31] and are presented as neutral detergent fiber (NDF), acid detergent fiber (ADF), lignin (ADL), cellulose (ADC), and hemicellulose fractions (Fibertec, Foss Tecator, Höganäs, Sweden).

### 2.6. Calcium Analysis in the Diets

One gram samples of the diets were ashed in a muffle furnace at 450 °C until complete mineralization was achieved, and then dissolved in 1 mol/L nitric acid (Merck, Kenilworth, NJ, USA). The mineral content of the samples was determined using flame atomic absorption spectrometry (AAS-3, Carl Zeiss, Jena, Germany) after appropriate dilution with deionized water and LaCl3 (0.5%). The methods were validated by a simultaneous analysis of the reference material (Brown Bread BCR191, Sigma-Aldrich, St. Louis, MO, USA), with an accuracy of 92%.

### 2.7. Calcium Analysis in the Tissues

Each sample was mineralized in a Microwave Digestion system (Speedwave Xpert, Berghof, Eningen, Germany) by digesting in 65% (*w*/*w*) spectra pure HNO_3_ (Merck, Kenilworth, NJ, USA). Deionized water was added and mixed after the digestion process. The mineral concentrations of the solutions were measured using flame atomic absorption spectrometry (AAS-3, Carl Zeiss, Jena, Germany) after appropriate dilution with deionized water and LaCl_3_ (0.5%) at a wavelength of λ = 422.7 nm. Verification used certified reference materials (Bovine liver 1577C, Sigma-Aldrich, Saint Louis, MO, USA) to determine the method’s accuracy, which was found to be 91% for Ca.

### 2.8. Serum Parameters

Serum concentrations of PINP, PTH, ES, and OC were determined with ELISA and commercial kits (SunRed, Shanghai, China). Spectrometry was performed using an Infinite F50 spectrometer (Tecan Group Ltd., Männedorf, Switzerland).

### 2.9. Statistical Analysis

All results are presented as the means ± standard deviations. Statistical analyses were performed using Statistica (StatSoft, Tulsa, OK, USA). The Shapiro–Wilk test was used to determine the normality of the variable distributions. Statistical differences between the groups were assessed using one-way ANOVA followed by Tukey’s post hoc test for normal distribution of values. However, Student’s *t*-test was used for comparing two groups. A *p*-value < 0.05 was considered statistically significant.

## 3. Results

The diet compositions are presented in Table 1. The CaL_B and P_CaL_B diets were equivalent to the CaL and P_CaL diets and had the same nutritional composition. The higher content of carbohydrates in the diet of the control group and OVX_C was probably due to the higher content of cornstarch (cornstarch was replaced with calcium lactate and pumpkin in modified diets).

We determined the effects of the modified diets on selected parameters. The results of the intervention are presented in Table 2, Table 3, Table 4 and Table 5.

Table 2 shows the daily intake of diet and calcium and the results of body composition analysis. The daily intake was comparable in all groups. Ovariectomy significantly increased the bodyweight and fat content of the rats. It was observed that groups that received enriched pumpkin (P_CaL and P_CaL_B) showed significantly decreased fat content, compared to the other ovariectomized groups.

All the ovariectomized groups showed a significantly lower serum ES concentration compared to the control group, which was not altered by the addition of calcium lactate or enriched pumpkin (Table 3). The obtained estrogen levels in groups confirmed the decreased concentration of estrogen after ovariectomy.

Table 4 presents the calcium concentrations in the serum and tissues by experimental group. When all groups were compared, we observed that ovariectomy did not affect the serum calcium concentration, but that the P_CaL group had a significantly higher calcium level than did the CaL group. Ovariectomy slightly decreased the calcium content in femurs, while the group receiving enriched pumpkin and alendronate (P_CaL_B) showed a significant increase in calcium content. It was found that ovariectomy significantly decreased calcium concentration in the heart and hair in rats, and that this effect was intensified by the other modified diets. Ovariectomy did not significantly affect the calcium content of the spleen or the liver. Calcium lactate markedly increased the concentration of calcium in the spleen, and the addition of the drug significantly lowered the concentration of this parameter in comparison to other groups. The enriched pumpkin significantly reduced the calcium content of the liver, while rats with alendronate had the highest levels of calcium in the liver of any of the groups. While ovariectomy did not alter muscle calcium content, calcium lactate and enriched pumpkin reduced it significantly, and the addition of the drug intensified this effect. Ovariectomy did not affect the calcium content of the kidneys. Groups P_CaL, CaL_B, and P_CaL_B showed significantly higher calcium levels in the kidney than did other groups. Enriched pumpkin more than doubled the calcium level in kidneys in comparison to the C and OVX_C groups. Moreover, alendronate increased the calcium level in kidneys by a factor of more than six, compared with the OVX_C group. In this study, we compared the OVX_C and CaL groups, which differed by the addition of calcium salt to the standard diet. Calcium lactate in the standard diet (CaL) significantly decreased calcium in the hair and muscle while increasing it in the spleen and kidneys, compared to the OVX_C group. Rats receiving the enriched pumpkin diet (P_CaL) had significantly higher calcium levels in the hair and kidney and markedly lower calcium levels in the spleen and liver than did the CaL group. The addition of alendronate to both the calcium lactate diet and to the enriched pumpkin diet increased the calcium level in femurs (although markedly only in the P_Ca_B group), livers, and kidneys, while decreasing calcium level in hearts and muscles, as compared to the CaL and P_CaL groups.

Table 5 shows the levels of the parameters involved in calcium and bone metabolism. We observed that ovariectomy increased the serum concentration of PINP. On the other hand, the modified diets led to a decrease in the serum PINP concentration, except for in the P_CaL_B group. Ovariectomy decreased serum PTH concentration, while all the modified diets increased it to values similar to those found in the control group. Ovariectomy, calcium lactate, and enriched pumpkin did not significantly affect the level of OC, while the addition of the drug reduced it, compared to the control group.

## 4. Discussion

It was generally observed that, in the femurs and kidneys of the rats fed fortified pumpkin, the levels of calcium were high, while most of the other organs (pancreas, spleen, heart, and muscles) contained low levels of the mineral. This may indicate a shift of calcium ions from other tissues to the bones, mostly to the kidneys, with consequent accumulation. We observed, in the P_CaL_B group, a significant decrease in thigh muscle calcium, while femur calcium was significantly higher than in the OVX_C group. This may indicate a shift of calcium from the thigh muscle to the bone attached to this muscle, balancing bone calcium content. Interactions between muscles and bones have been confirmed by previous studies showing that the communication between these is not only mechanical, but also acts through the secretion of biochemical factors and minerals [32,33,34]. The results of those studies confirmed that alendronate inhibits bone resorption in the postmenopausal condition and reduces the loss of calcium from bone, at the expense of decreasing calcium in other organs [35,36]. A probable mechanism of the increased calcium content in the tissues after the consumption of fortified pumpkin is enhanced absorption of calcium by inulin, which was a component of the enriched pumpkin. The large influx of ions into the body is then distributed to the tissues bearing the CaSR (calcium-sensing receptor) cells. Through this receptor, calcium ions from the external environment pass into the internal, thus increasing the calcium content in the tissues [12,37]. The change in tissue distribution of calcium ions was likely the reason for the observed low levels of calcium in the liver in the enriched pumpkin group compared to the calcium lactate group. The group enriched with pumpkin had a relatively low concentration in the liver and pancreas, but a high calcium content in the kidneys, which may indicate a shift of calcium ions and its increased excretion and increased reabsorption in the kidneys.

However, in the present study, we saw both beneficial and detrimental effects of pumpkin enrichment on the calcium status of ovariectomized rats. The greatly increasing calcium concentration in the kidneys with the consumption of enriched pumpkin was unexpected and could lead to serious complications in the body. Calcium accumulation in the kidneys may be associated with increased excretion of this mineral, although, in this study, the calcium concentration of the animal’s urine was not considered; this may limit the clear interpretation of the phenomenon. Our results may indicate possible side-effects of the components of the calcium-enriched pumpkin, such as inulin which, despite increasing the absorption of calcium from the intestines [28], also contributes to its increased excretion [38]. Calcium accumulation in the kidneys can lead to kidney dysfunction, causing kidney stones [39] and calcification of soft tissues [10] and blood vessels [40]. The main components of kidney stones are calcium oxalate (approximately 80%) and calcium phosphate (about 15%). The consequences of untreated kidney stones may include inflammation of the urinary system and chronic kidney disease [41]. Nephrolithiasis is also a risk factor for osteoporotic fractures. The kidneys play an important role in the homeostasis of calcium, which is filtered and reabsorbed in this organ. If calcium is improperly filtered or reabsorbed, it causes hypercalciuria and a decrease in serum calcium, leading to the increased release of calcium from bones to the plasma. BMD consequently decreases, and osteoporosis can develop [42]. Some other components of pumpkin which may affect renal function are vitamins A and E [24]. Rats with pumpkin in their diet had definitely higher supply of vitamins A and E than did those receiving the standard diet, and excessive amounts of these vitamins can lead to glomerular hyperfiltration and hypercalcemia [43]. The biologically active form of vitamin A is retinoic acid, which can lead to the progression of glomerular disease [44,45]. However, in this study, the vitamin levels in blood and tissues were not measured; hence, the above explanation for the negative effects of fortified pumpkin on the kidneys is only speculation. In studies by other authors, pumpkin has a rather positive effect on the kidneys [46,47,48]. The present study may indicate that enriched pumpkin increases the concentration of calcium in the kidneys. It is possible that the glomeruli had become damaged by the excessive saturation of the body with vitamins, impairing calcium reabsorption. Similar differences were noted with respect to calcium concentration in the kidneys in the CaL group. Inulin is also responsible for the increased retention of calcium in the bones, which affects the accumulation of calcium, magnesium, and iron in the bones, but only when the demand for minerals is high, such as during growth [26].

Another component of the rats’ diet that affects calcium metabolism is alendronate, the main bisphosphonate drug. Alendronate is widely used in women with osteoporosis, where it is administered orally in weekly doses. There are many studies on the prevention of bone fractures in patients using alendronate [49]. Bisphosphonates, through coordinating between the calcium ions of the crystal structure and the phosphonate groups, bind specifically with hydroxyapatite [50]. Alendronate works by inhibiting bone resorption through the induction of osteoclast apoptosis. Alendronate also stimulates osteoblast differentiation and alleviates the apoptosis of osteoblasts and osteocytes [51]. However, bisphosphonates should not be used for long periods, as they have numerous side-effects, including kidney damage. The strong affinity of alendronate for calcium ions, soluble calcium, and insoluble calcium causes the formation of aggregates and complexes [52], which may be retained in the kidneys, damaging renal tubules and causing them to undergo necrosis [53]. Although nephrotoxicity is mainly an issue for intravenous bisphosphonates (oral medications usually do not show similar side-effects), there are some exceptions. In one study [54], significant increases in kidney calcium levels were observed in rats fed a diet supplemented with alendronate; this may indicate adverse calcium accumulation in the kidneys. We can only assume that there were changes in the kidneys of the rats (such as kidney stones and calcification) receiving enriched pumpkin or alendronate, as we unfortunately do not have results to support this speculation. In the future, we plan to extend the analysis to include the biochemical and histopathological parameters of kidney function. An association between natural menopause and surgical menopause with a higher risk of an incident kidney stone has been found in clinical studies [55]. It, thus, seems that calcium-enriched pumpkin, especially when combined with alendronate, could increase the risk of this kind of renal dysfunction in menopausal women.

We also observed an effect of the enriched pumpkin on the hormonal balance of calcium metabolism. Apart from obvious hormonal changes, such as the decrease in ES concentration after ovariectomy, an increase in PINP concentration after ovariectomy was also observed, which may indicate an increase in bone formation and, thus, bone turnover. On the other hand, in the groups with the modified diets, ovariectomy led to a smaller increase in PINP concentration. PINP is a marker of bone formation, and, in the absence of estrogen, its concentration increases, which leads to more pronounced bone turnover [56,57]. In our study, the modified diets produced an effect similar to that of administration of ES, except for in the P_CaL_B group. The opposite effect on PINP seen in the P_CAL_B group may be due to the interaction between alendronate and enriched pumpkin. This phenomenon requires further research. We moreover found that ovariectomy reduced the concentration of PTH and OC, while modified diets normalized the PTH level in the ovariectomized rats. It is known that PTH concentration depends on the amount of calcium in the blood. However, our results did not confirm this. Modified diets may have impacted other biochemical or hormonal factors associated with the experimental parameters.

Pumpkin enriched with calcium lactate also affects body composition. In a previous study, it was found that calcium lactate-enriched pumpkin decreased serum leptin levels, thereby lowering body fat and weight in ovariectomized rats [58].

### Limitations

This study had a number of limitations which may have affected the results. The study did not involve a sham group; therefore, the effect of sham surgery could not be taken into account. However, we did compare the effect of ovariectomy with an unoperated control group. We did not consider indicators of oxidative stress, antioxidant status, or vitamins. The composition of the diet was not analyzed in detail (e.g., vitamin D level was not measured). Moreover, the rats’ urine was not collected, and parameters of renal function were not noted; these might have broadened the interpretation and affected our discussion of the results.

## 5. Conclusions

Pumpkin enriched with calcium lactate affects calcium status and normalizes PINP and PTH serum levels in ovariectomized rats. A diet enriched with pumpkin and alendronate increases calcium concentration in the femur. Calcium lactate-enriched pumpkin causes calcium to accumulate in the kidneys of ovariectomized rats. Alendronate significantly exacerbates this adverse renal effect. Our results, thus, point to possible side-effects of using calcium lactate-enriched pumpkin.

## Figures and Tables

**Table 1:** The composition of the diets (mean and standard deviation).

Diet	Group		
	C/OVX_C	CaL/CaL_B	P_CaL/P_CaL_B
Caloric value (kcal/100 g)	326.37 ± 4.48	321.87 ± 3.37	322.27 ± 1.66
Carbohydrates (g/100 g)	47.92 ± 0.60 ^b^	44.53 ± 0.90 ^a^	45.44 ± 0.65 ^a^
Fiber (g/100 g)	23.04 ± 0.60	25.05 ± 1.60	23.02 ± 0.41
Fat (g/100 g)	3.76 ± 0.41	4.33 ± 0.16	4.27 ± 0.02
Protein (g/100 g)	13.70 ± 0.21	13.67 ± 0.81	14.01 ± 0.59
Calcium (mg/g)	5.63 ± 0.37	5.68 ± 0.24	5.77 ± 0.15

C, control group; OVX_C, ovariectomized group; CaL, ovariectomized group with calcium lactate; CaL_B, ovariectomized group with calcium lactate and alendronate; P_CaL, ovariectomized group with pumpkin enriched with calcium lactate; P_CaL_B, ovariectomized group with pumpkin enriched with calcium lactate and alendronate; ^a,b^ significant differences between groups (*p* < 0.05).

**Table 2:** Daily intake and parameters of the body composition analysis in rats (mean and standard deviation).

Parameter	Group					
	C	OVX_C	CaL	P_CaL	CaL_B	P_CaL_B
Daily intake of diet (g)	25.08 ± 0.63	25.11 ± 1.70	25.90 ± 0.55	24.31 ± 1.26	25.66 ± 2.29	24.84 ± 2.29
Daily intake of calcium (mg)	141.12 ± 3.56	141.30 ± 9.57	147.03 ± 3.11	140.31 ± 7.26	139.73 ± 12.44	145.01 ± 13.39
Body mass (g)	325.86 ± 25.97 ^a^	421.90 ± 55.10 ^b^	428.40 ± 51.1 ^b^	384.11 ± 34.02 ^ab^	433.30 ± 51.62 ^b^	392.30 ± 34.88 ^b^
Fat tissue (g)	122.08 ± 35.78 ^a^	238.66 ± 55.07 ^bc^	252.27 ± 47.47 ^c^	167.79 ± 31.34 ^ab^	267.13 ± 77.16 ^c^	182.14 ± 37.70 ^ab^

C, control group; OVX_C, ovariectomized group; CaL, ovariectomized group with calcium lactate; CaL_B, ovariectomized group with calcium lactate and alendronate; P_CaL, ovariectomized group with pumpkin enriched with calcium lactate; P_CaL_B, ovariectomized group with pumpkin enriched with calcium lactate and alendronate; ^a,b,c^ significant differences between groups (*p* < 0.05).

**Table 3:** The level of estradiol in serum of rats (mean and standard deviation).

Parameter	Group					
	C	OVX_C	CaL	P_CaL	CaL_B	P_CaL_B
ES ng/L	49.82 ± 4.62 ^b^	21.68 ± 6.80 ^a^	23.35 ± 2.58 ^a^	20.05 ± 6.54 ^a^	19.85 ± 2.50 ^a^	18.12 ± 4.29 ^a^

C, control group; OVX_C, ovariectomized group; CaL, ovariectomized group with calcium lactate; CaL_B, ovariectomized group with calcium lactate and alendronate; P_CaL, ovariectomized group with pumpkin enriched with calcium lactate; P_CaL_B, ovariectomized group with pumpkin enriched with calcium lactate and alendronate; ES—estradiol; ^a,b^ significant differences between groups (*p* < 0.05).

**Table 4:** Calcium content in the serum and tissues (mean and standard deviation).

Tissue	Group					
	C	OVX_C	CaL	P_CaL	CaL_B	P_CaL_B
Serum (ug/mL)	133.04 ± 11.91 ^ab^	121.80 ± 8.10 ^ab^	115.58 ± 6.23 ^a^	139.10 ± 9.84 ^b^	122.89 ± 12.99 ^ab^	127.51 ± 17.27 ^ab^
Femur (mg/g dm)	234.53 ± 21.67 ^ab^	217.48 ± 7.24 ^a^	231.98 ± 60.74 ^ab^	235.80 ± 13.37 ^ab^	254.54 ± 19.15 ^ab^	267.63 ± 23.63 ^b^
Pancreas (ug/g dm)	108.81 ± 11.46	113.75 ± 10.03	128.72 ± 19.30	106.66 ± 26.70	116.85 ± 11.69	107.56 ± 21.92
Hair (ug/g dm)	574.21± 75.87 ^d^	459.45 ± 50.19 ^c,#^	304.27 ± 39.59 ^a^	382.73 ± 49.75 ^b,#^	389.72 ± 49.98 ^b^	382.41 ± 23.11 ^b^
Spleen (ug/g dm)	530.71 ± 109.98 ^bc^	470.30 ± 99.57 ^b,#^	592.83 ± 39.82 ^c^	450.19 ± 89.14 ^b,#^	291.36 ± 46.23 ^a,#^	316.06 ± 44.92 ^a,^*
Liver (ug/g dm)	158.08 ± 10.47 ^cd^	139.58 ± 11.83 ^bc^	132.82 ± 16.54 ^ab^	117.59 ± 9.25 ^a,#^	161.20 ± 19.83 ^d,#^	169.85 ± 11.64 ^d,^*
Heart (ug/g dm)	119.67 ± 14.73 ^c^	81.90 ± 19.50 ^b^	80.44 ± 12.5 ^b^	75.93 ± 8.01 ^b^	75.05 ± 7.31 ^b^	53.80 ± 6.63 ^a,^*
Brain (ug/g dm)	182.33 ± 31.12	221.45 ± 81.06	177.15 ± 78.82	219.57 ± 97.63	209.70 ± 77.67	181.34 ± 28.7
Muscle (ug/g dm)	49.13 ± 8.25 ^cd^	52.23 ± 10.86 ^d,#^	36.25 ± 10.35 ^bc^	34.88 ± 9.30 ^b^	21.58 ± 9.68 ^a,#^	18.67 ± 5.82 ^a,^*
Kidney (ug/g dm)	87.88 ± 12.42 ^a^	79.29 ± 7.66 ^a,#^	123.92 ± 23.58 ^a^	194.13 ± 41.01 ^b,#^	477.78 ± 80.4 ^c,#^	541.33 ± 62.91 ^d,^*

C, control group; OVX_C, ovariectomized group; CaL, ovariectomized group with calcium lactate; CaL_B, ovariectomized group with calcium lactate and alendronate; P_CaL, ovariectomized group with pumpkin enriched with calcium lactate; P_CaL_B, ovariectomized group with pumpkin enriched with calcium lactate and alendronate; dm, dry mass; ^a,b,c,d^ significant differences between groups (*p* < 0.05); # significantly different in comparison to Ca_L group; * significantly different in comparison to P_CaL group.

**Table 5:** Parameters of calcium and bone metabolism in serum of rats (mean and standard deviation).

Parameter	Group					
	C	OVX_C	CaL	P_CaL	CaL_B	P_CaL_B
PINP (ng/mL)	3.25 ± 1.10 ^a^	4.69 ± 0.82 ^c^	4.45 ± 0.82 ^ab^	4.14 ± 0.69 ^ab^	3.77 ± 0.33 ^ab^	4.62 ± 0.54 ^c,^*
PTH (ng/dL)	3.35 ± 0.5 ^b^	2.59 ± 0.45 ^a,#^	3.39 ± 0.54 ^b^	3.38 ± 0.57 ^b^	3.47 ± 0.28 ^b^	2.80 ± 0.35 ^ab^
OC (ng/mL)	18.91 ± 3.59 ^b^	16.23 ± 1.08 ^ab^	15.61 ± 3.38 ^ab^	14.53 ± 4.40 ^ab^	12.78 ± 4.70 ^a^	11.70 ± 3.04 ^a^

C, control group; OVX_C, ovariectomized group; CaL, ovariectomized group with calcium lactate; CaL_B, ovariectomized group with calcium lactate and alendronate; P_CaL, ovariectomized group with pumpkin enriched with calcium lactate; P_CaL_B, ovariectomized group with pumpkin enriched with calcium lactate and alendronate; PINP, procollagen type I N propeptide; PTH, parathyroid hormone; OC, osteocalcin; ^a,b,c^ significant differences between groups (*p* < 0.05); # significantly different in comparison to Ca_L group; * significantly different in comparison to P_CaL group.

## Data Availability

The data used to support the findings of this study can be made available by the corresponding author upon request.
